# Water stable isotope data set in temperate, lowland catchment, two years of monthly observations, River Salaca, Latvia

**DOI:** 10.1016/j.dib.2020.105607

**Published:** 2020-04-22

**Authors:** Andis Kalvāns, Alise Babre, Aija Dēliņa, Konrāds Popovs

**Affiliations:** University of Latvia, Faculty of Geography and Earth Sciences, Jelgavas str. 1., Riga, Latvia, Lv-1004

**Keywords:** Water stable isotopes, Hydrogeology, Catchment, Evaporation, Groundwater-surface water interaction

## Abstract

Two years of monthly observations of water stable isotopes in a temperate lowland catchment with extensive agrarian and forested landscapes in Latvia, River Salaca catchment. Observations include most significant water types within catchment: precipitation, raised bog, intermittent, groundwater and surface water at 15 observation points. The monthly data is supplemented by two intervals of one-month long sampling every second day for a subset of observation points. Water table, temperature and electrical conductivity co-observed as key variables helping to understand the isotope data. The data can be useful for in depth investigation of isotope hydrology and as background information for ecohydrological and other studies. The data is associated with the original research article “An insight into water stable isotope signatures in temperate catchment” [1].

## Highlights

• Two years of monthly water stable isotope ratios from temperate lowland catchment.
• Hourly water table, temperature and electrical conductivity monitoring.
• Distinct isotope ratios for raised bog, groundwater and rivers.

Specifications Table

**Table:** 

Subject	Earth-Surface Processes
Specific subject area	Stable isotope hydrology, surface water – groundwater interaction
Type of data	Table Graph
How data were acquired	Water samples collected in the field, ratios of water stable isotopes measured by Picarro L2120-i Isotopic Water Analyzer
Data format	Raw
Parameters for data collection	Dominant water types in a temperate (hemi boreal) lowland catchment were sampled
Description of data collection	Staring from August 2015 for 25 consecutive month one water sample each month at 15 observations points were collected that include major river and its tributaries, raised bog, groundwater and precipitation. Ratios of water stable isotopes expressed as δ^18^O and δ^2^H were measured with Cavity Ring-Down Spectroscopy. In addition, water table and electrical conductivity were measured with automatic probes with one-hour intervals at certain location.
Data source location	Latvia, River Salaca catchment: N 57.796° to N 57.945° latitude, E 24.792° to E 25.142° longitude
Data accessibility	With the article
Related research article	Kalvāns, A., Dēliņa, A., Babre, A., Popovs, K. 2020. An insight into water stable isotope signatures in temperate catchment. *Journal of Hydrology*, https://doi.org/10.1016/j.jhydrol.2019.124442[1]

## Value of the Data

• Presented 25-month time series provide unique insight into dynamics and seasonality of stable isotope ratios in different water types within temperate lowland catchment.
• Researchers working on hydrology related problems can used the data set as background information in planning their investigation or as input for global simulations of water using cycle using natural isotope tracer methods
• The data set can be used as input for compiling high resolution or temporal isoscapes of groundwater, wetlands, surface water and biota and to examine the secular variations of stable isotope ratios as water transitions between compartment of terrestrial hydrological cycle

## 1 Data Description

**Fig. 1:**
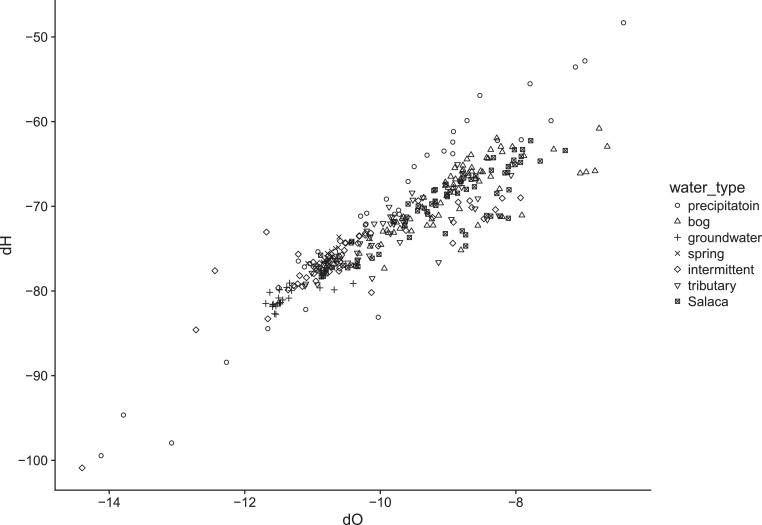
Dual isotope plots of monthly isotope ratios in River Salaca catchment

We share a time series from August 2015 to August 2017 of monthly water stable isotope ratios – δ^18^O and δ^2^H (Figure 1, Data set – 1) at 15 sampling locations (Table 1) from Latvia, River Salaca catchment. The data encompasses 640 individual water stable isotope analyses, but 88 of those were flagged as unreliable due to contamination. Isotope ratios are complemented with water table and electrical conductivity observations (Data set – 2) as indicated in Table 1. Geolocation of the sampling points is available in Supplementary material – 1.

**Table 1:** Summary of the sampling points.

Site code	Description	Automatic probes	N Lat. / E Long. / elevation m a.s.l.	Classification
SV1	River Salaca near its outlet from the Lake Burtnieks	L	57.79619 / 25.14168 / 45.41	Salaca River
SV2	River Salacabetween SV1 and Sv3 sampling points	-	57.89403 / 25.00277 / 37.4*	
SV3	River Salaca downstream of two major tributaries, Ramata and Ige	L, EC	57.85667/ 24.79163 / 40.23	
RV1	River Ramata, tributary to the River Salaca	L, EC	57.93623 / 25.00593 / 48.65	Tributaries
IV1	River Ige, tributary to the River Salaca	L, EC	57.89210 / 24.88635 / 49.31	
PP	Small river (Piģele) draining a raised bog and lake, within a bog	L	57.94523 / 24.90668 / 53*	Raised bog
PV1	Small river (Piģele) draining a raised bog after emergence from it	-	57.92968 / 24.89915 / 50.16	
LU1	Shallow well in raised bog; filter interval opening 0.10 to 0.90 m depths below surface	L	57.94523 / 24.90675 / 53*	
GU1	Shallow well into siltstones with sandy interbeds of the D_2_Burnieks Formation; filter depth 3.7-4.7 m	L	57.89403 / 25.00277 / 38*	Groundwater
GA1	Govs Ala natural spring	-	57.89403 / 25.00277 / 39*	
RU1	Shallow well, glacial till in an agricultural land with tile drainage; filter depth 3.13 to 4.13 m, opening to sand-gravel interbed	L	57.93552 / 24.98158 / 47.91	Intermittent water and phreatic groundwater, fine grained soils
RU2	Shallow well in sand to clay slope deposits at the slope of the River Ramata valley flanking agricultural land with tile drainage; filter depth 2.27 to 3.27 m	-	57.93346 / 24.98131 / 42.17	
RU3	Outlet of the tile drainage on arable land on loam soil	L, EC	57.9346 / 24.9775 / 44.04	
RN1	Precipitation sampled in line with guidelines issued by IAEA [2].	-	57.93433/ 24.98035 / 46.30	Precipitation
RN2		-	57.93500 / 24.98241 / 47.25	

L – automated relative water table measurements

EC – automated electrical conductivity measurements

⁎ elevation determined from 1:10 000 or 1:25 000 topographic maps, in other cases high accuracy GPS station is used

## 2 Experimental Design, Materials, and Methods

Data set covers observations along River Salaca located in North Eastern part of Latvia with temperate continental climate in hemiboreal vegetation zone. The lowland catchment is characterised by patchwork of extensive agricultural lands, coniferous forests, raised bogs and small settlements. River Salaca is an outflow of 40 km^2^ large Lake Burtnieks, that is a flow lake with water turnover 6 to 7 times a year, 2-3 weeks during the spring and 3 months during the summer [3]. The catchment area of the lake is 2215 km^2^. The lake has several small tributaries. Modelled groundwater head distribution indicate that groundwater discharge in the lake was taking place [4]. Several rivers as and groundwater is discharging in the lake. Fifteen separate water sampling points were established covering water in precipitation, raised bog, intermittent, groundwater and surface water (Table 1, see refs. [1,5] for detailed description).

Stable isotope ratios in water were analysed in the Laboratory of Environmental Dating at the Faculty of Geography and Earth sciences, University of Latvia. The results are expressed in standard δ-notation relative to Vienna Standard Mean Ocean Water (VSMOW; [6]). Cavity ring-down laser spectroscopy method [7] with Picarro L2120-i Isotopic Water Analyzer was used, following procedures elaborated by IAEA [8]. The measurement reproducibility is ±1 ‰ for δ^2^H and ±0.1 ‰ for δ^18^O. The Laboratory has successfully participated in the water isotope laboratory proficiency tests [9].

Samples were collected monthly for 25 consecutive months starting from August 2015 (Figure 1). At selected sampling points for July 2016 and November 2016 samples were collected every second day. These intervals correspond to midsummer with expected highest temperatures and late autumn with expected groundwater recharge. All isotope results were subject to quality check for consistency. If during sample collection irregularities occurred, such as contamination with other water sources or blockage of tubing in precipitation traps were observed, isotope results were discarded. If observation had strong deviation from average range at the sampling location, hydro-meteorological conditions were scrutinised. Then it was decided upon expert judgement (the authors) if measured value was realistic. If strong discrepancies between results from two precipitation traps were found, either both of the measurements or the measurement showing irregularities such as influence of evaporation or problems with sampling setup were labelled “unreliable”. After a quality check 88 observations were labelled as “Unreliable” (Data set - 1) due to unrealistic and inconsistent values. Electrical conductivity (EC) and pH were measured in field. Samples were collected in 25 mL HDPE bottles without filtration. During transport and storage samples were kept refrigerated (<4°C) until analysis.

Water table, temperature and electrical conductivity was measured with automatic probes (Diver, vanEssen Instruments) at selected locations (see Table 1 and attached Interactive Map Data). Accumulated precipitation water volume was measured in the field, any snow collected on the precipitation traps was collected and added to the precipitation sample.

## Appendix Supplementary materials

Image, application 1

Image, image 2

Image, image 2
